# Experimental Investigation and Optimization of Turning Polymers Using RSM, GA, Hybrid FFD-GA, and MOGA Methods

**DOI:** 10.3390/polym14173585

**Published:** 2022-08-30

**Authors:** Abdulrahman I. Alateyah, Yasmine El-Taybany, Samar El-Sanabary, Waleed H. El-Garaihy, Hanan Kouta

**Affiliations:** 1 Department of Mechanical Engineering, College of Engineering, Qassim University, Unaizah 56452, Saudi Arabia; 2 Department of Production Engineering and Mechanical Design, Port Said University, Port Fouad 42526, Egypt; 3 Mechanical Engineering Department, Faculty of Engineering, Suez Canal University, Ismailia 41522, Egypt

**Keywords:** polymers, turning, surface roughness, MRR, chip formation, ANOVA, optimization, RSM, GA, MOGA

## Abstract

The machining of polymers has become widely common in several components of industry 4.0 technology, i.e., mechanical and structural components and chemical and medical instruments, due to their unique characteristics such as: being strong and light-weight with high stiffness, chemical resistance, and heat and electricity insolation. Along with their properties, there is a need to attain a higher quality surface finish of machined parts. Therefore, this research concerns an experimental and analytical study dealing with the effect of process parameters on process performance during the turning two different types of polymers: high-density polyethylene (HDPE) and unreinforced polyamide (PA6). Firstly, the machining output responses (surface roughness (Ra), material removal rate (MRR), and chip formation (λc)) are experimentally investigated by varying cutting speed (v_c_), feed rate (f), and depth of cut (d) using the full factorial design of experiments (FFD). The second step concerns the statistical analysis of the input parameters’ effect on the output responses based on the analysis of variance and 3D response surface plots. The last step is the application of the RSM desirability function, genetic algorithm (GA), and hybrid FFD-GA techniques to determine the optimum cutting conditions of each output response. The lowest surface roughness for HDPE was obtained at v_c_ = 50 m/min, f = 0.01 mm/rev, and d = 1.47 mm and for PA6 it was obtained at v_c_ = 50 m/min, f = 0.01 mm/rev, and d = 1 mm. The highest material removal rate was obtained at v_c_ = 150 m/min, f = 0.01 mm/rev, and d = 1.5 mm for both materials. At f = 0.01 mm/rev, d = 1.5 mm, and v_c_ = 100 for HDPE, and v_c_ = 77 m/min for PA6, the largest chip thickness ratios were obtained. Finally, the multi-objective genetic algorithm (MOGA) methodology was used and compared.

## 1. Introduction

Nowadays, the demand for polymers in several industries and manufacturing areas, especially industry 4.0 technology, has increased owing to their unique physical, mechanical, and thermal properties. High specific strength and modulus, good damping properties, low density and weight, good corrosion resistance, mass production possibility, low friction coefficient, good thermal/electrical insulation, and the ability to be processed without external lubrication are the main advantages of polymers over metallic materials. As a result of these exceptional properties, polymers are used in numerous applications such as gears, bearings, rolling elements, structural components, and chemical and medical instruments [1,2,3,4]. However, polymers are considered as difficult-to-cut materials as they have few limitations over metals and alloys, such as the melting point of polymers being comparatively low, which leads to the easy softening of polymers when machining due to heat generated in the working area. Therefore, the applications of polymers at high working temperatures is not preferred [5]. In addition, the thermal expansion of polymers is ten times more than metals, which is one of the limitations that need to be considered in some applications [6].

Generally, primary injection molding processes i.e., injection, blow, compression, transfer molding, etc., are used for the manufacturing of large-scale polymeric products [7]. However, these methods can be somewhat restrictive and do not produce good dimensional accuracy or surface finish requirements. Thus, machining and post-processing are preferred in these cases [8,9,10]. High dimensional accuracy and superior surface quality are vital requirements of polymers products in precision machinery, electronics, medical, and optics applications. To attain these characteristics, polymers need to undergo machining operations such as turning, milling, and drilling [5,6,7].

Therefore, the machining of polymers has become the focus of interest for many researchers and many attempts have been made for the sake of understanding the polymers’ performance and gaining knowledge of their machinability characteristics as well as obtaining the best conditions during machining operations using different statistical analysis tools.

Correa et al. [11] analyzed the effect of cutting speed, feed rate, and tool tip angle on the holes’ dimensional deviations, circularity error, fiber pull out, and burr formation during the drilling of polyamide reinforced with 25% glass fiber. Kannan et al. [12] investigated the hole quality and thrust force during drilling of carbon fiber reinforced polymers (CFRPs). Gaitonde et al. [13] examined the surface roughness in the high-speed drilling of unreinforced polyamide (PA6) and reinforced polyamide with 30% glass fibers (PA66 GF30) by developing response surface methodology. Solymani et al. [14] conducted numerous drilling experiments on PA6 nanocomposites to obtain the thrust force values, used a particle-swarm-optimization-based neural network (PSONN) to create a predictive model, and compared the results with that of the conventional neural network.

To understand the micro-machinability of polyamide-6 (PA6) and glass-fiber-reinforced polyamide 6 (GFPA6), Kuram et al. [15] investigated the influences of various spindle speeds and feed rates on the cutting force, surface roughness, burr formation, and tool wear during the micro-milling of these materials. Other different kinds of polymers such as polymethylmethacrylate (PMMA), polyetheretherke tone (PEEK), and polyimide (PI) were experimentally tested in terms of surface roughness, burrs, and cutting chip characteristics in the high-speed micro-milling process [16]. From the micro-machinability point of view, better results were obtained from unreinforced PA6 compared to GFPA 6. Surface roughness was experimentally investigated and optimized and a predictive model based on an artificial neural network (ANN) was developed during the milling of (PA-6) nanocomposites [17] and slot milling of polypropylene materials [18]. In addition, the response surface methodology (RSM) technique was used to evaluate the effect of machining parameters on surface roughness during the milling of polyetheretherketones (PEEK) implant material [19]. Based on their optimization results, a better surface finish was obtained at the lowest level of feed rate. Verma et al. [20] applied a hybrid method during the milling of epoxy nanocomposites reinforced by graphene oxide/carbon fiber (G/CF) to optimize the material removal rate (MRR), cutting force (Fc), and surface roughness (Ra).

Other attempts were made to develop a model for surface roughness prediction and optimization when turning PA6 by applying RSM and analysis of variance (ANOVA) to the experimental data [21], as well as an ANN [22], and the fuzzy logic technique along with the Taguchi’s orthogonal array [2]. Machinability aspects in terms of cutting force, power, and specific cutting pressure, when turning unreinforced (PA6) and reinforced (PA66 GF30) polyamides [23] and PEEK, unreinforced and reinforced (GF30), ref [24] using the RSM-based parametric analysis results, revealed that cutting force and power increased with cutting conditions, whereas specific cutting pressure decreased by increasing the feed rate. In the same context, Vaxev et al. [25] studied the influence of cutting speed and feed rate on cutting force components during the turning of PA66 GF30 by application of ANOVA and ANN. Aldwell et al. [26] analyzed the effects of turning parameters on the chip formation, cutting forces, and surface roughness in ultra-high-molecular-weight polyethylene, a common material used in biomedical applications. M. Kaddeche et al. [27] evaluated the surface roughness, cutting pressures, and temperatures during the turning of two grades of high density polyethylene (HDPE-100 and HDPE-80) extruded pipes and ANOVA was performed to establish predictive models. In addition, Hamlaoui et al. [28] experimentally investigated the influence of cutting speed, feed rate, and depth of cut on surface roughness and cutting temperature during the turning of tough polyethylene pipe material HDPE-100 using a Taguchi (L27) orthogonal array. A predictive model was established between input and output parameters via the RSM. The material removal rate was optimized along with different combinations of the process control parameters, such as the cutting speed, feed rate, and depth of cut using the Taguchi design and ANOVA methods during the turning of Teflon (PTFE) cylindrical rounds [29]. Chabbi et al. [30] applied the RSM and ANN methods when turning polyoxymethylene (POM C) polymer in order to investigate surface roughness, cutting force, and power. In addition, optimizations were accomplished using the desirability function (DF).

**Figure 1:**
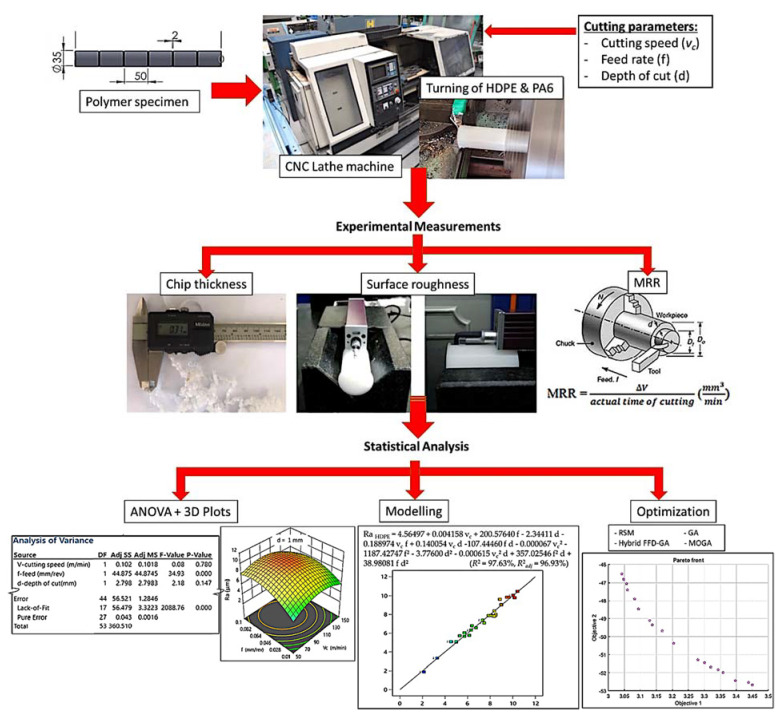
Flow chart of the experimentation strategy.

From the literature, limited work has been carried out to study the machinability aspects of polymers. Further, the relationship between the influencing factors and their effects on machinability are still not well-known. Among polymers, polyethylene (PE) and polyamides (PA) have been some of the most widely studied materials in polymer science due to their large number of applications. High density polyethylene (HDPE) pipes have been commonly used in recent urban networks for water conveyance due to their advantages in terms of setting up, joining, toxicological safety, and service lifetime [27]. Polyamides are used in applications where toughness, lubricity, and wear resistance are important, for example, in aircrafts, machines, marine applications, and automobile engine components such as sprockets, bearings, and gears [15]. Thus, this article aims at investigating, experimentally, the effects of cutting speed, feed rate, and depth of cut on surface roughness (Ra), material removal rate (MRR), and chip formation (λc) during the turning of two types of polymers: high-density polyethylene (HDPE) and unreinforced polyamide (PA6). These investigations are based on the concept of the full factorial design of experiment (FFD), which makes the experimental work not only effective but also efficient. Then, ANOVA and RSM are executed to analyze the process performance. After that, optimum cutting conditions are determined using genetic algorithm (GA) and hybrid FFD-GA approaches. A multi-objective genetic algorithm (MOGA) is used and compared as well. Figure 1 illustrates the flow of the experimentation strategy during this research.

## 2. Experimental Procedures

### 2.1. Materials and Measurement Methods

**Figure 2:**
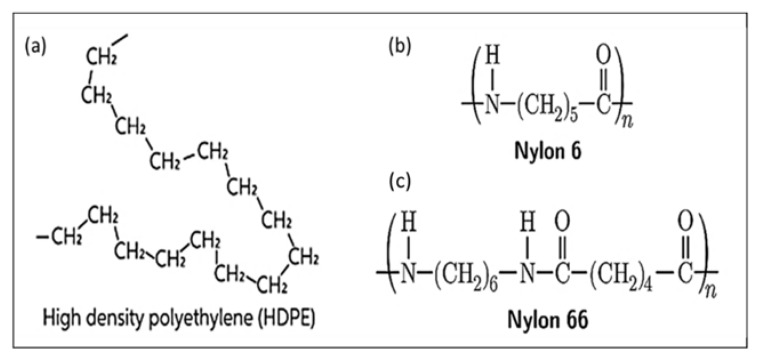
General structure of (**a**) Monomer ethylene, (**b**) PA6, and (**c**) PA66.

Two types of polymers, high-density polyethylene (HDPE) and Polyamides 6 (Nylon-pa), were used for the turning process. High-density polyethylene (HDPE) is a thermoplastic polymer produced from the monomer ethylene, as shown in Figure 2a. It is sometimes called “alkathene” or “polythene” when used for HDPE pipes with a high strength-to-density ratio. The Polyamides or Nylon class is one of the major engineering and high-performance thermoplastics classes because of its good balance of properties [28]. Polyamides contain repeating amide linkages (–CO-NH–), as shown in Figure 2b; they are formed by condensing identical units, copolymers, for different units. The two most common grades of Nylon are Nylon 6 (or PA6) and Nylon 66 (or PA66). The number relates to the number of methyl groups that are located on each side of the nitrogen atoms (amide groups). The main properties of these materials are given in Table 1. The workpiece specimens were cylindrical rods with diameters of 30 mm for HDPE and 35 mm for PA6, which were 300 mm in length and divided into six segments of 50 mm each, separated by 2 mm grooves.

A Colchester 4000L CNC lathe machine was used with Fanuc OT commands. Dry tests were carried out using a cemented carbide insert (K10) tool (DCMT 11 T3 02-MF 1125) bolted on a tool holder SDJCR1616k11/L of size 16 × 16 mm. In the present study, the arithmetic mean roughness (Ra) were selected to express the surface roughness, since it is the most widely used surface roughness parameter in the industry to judge the surface quality. The surface roughness (Ra) was measured by means of a Mitutoyo Surftest SJ-201 roughness meter. The specimens were held on a V-block while a spirit level was used to ensure the proper alignment of the stylus motion. The measurements were conducted at four different points in the longitudinal feed direction with a 2.5 mm cut-off and 12.5 mm evaluation length; then, the average of arithmetic mean value (Ra) was calculated to represent the surface roughness. Actual MRR was calculated by subtracting the final volume of the specimen after turning (mm^3^) from the initial volume of the specimen, then dividing it by the actual machining time (min). The chip thickness ratio (λc) is determined experimentally as the ratio between the deformed chip thickness (t_2_), which was measured using an electronic LCD digital vernier caliper with an accuracy of 0.01 mm, and the undeformed chip thickness (t_1_ = f·sinϕ), where f is the feed rate (mm/rev) and ϕ is the tool principal angle = 93°. The chips produced during turning were collected and the average deformed chip thickness was obtained by the average of ten readings at different locations of the same chips produced under constant conditions.

### 2.2. Experimentation and Data Collection

The turning experiments were planned using the full factorial design of experiments (FFD) consisting of three factors: cutting speed, feed rate, and depth of cut, with three levels, each with two replications, which led to a total number of 54 experiments. The values chosen were as follows: cutting speed v_c_ (50, 100, and 150 m/min), feed rate f (0.01, 0.05, and 0.1 mm/rev), and depth of cut d (0.5, 1, and 1.5 mm). Stat-Ease Design Expert (version 13.0.5) was used to generate the testing order as well as to assist in the statistical analysis of the experimental data. The experimental plan of the present investigation, as per FFD and their corresponding measured responses, is presented in Table 2.

Based on the experimental results, RSM was applied to analyze the results statistically to formulate the model, analyze, and develop an appropriate interaction between measured responses and input factors by regression modelling [31,32]. Regression models were obtained by the best interaction correlation between the input variables and the output responses into a non-linear equation. Finally, the optimal cutting conditions of turning HDPE and PA6 for the desired responses were attained by an application of the RSM desirability function, a genetic algorithm (GA), and hybrid FFD-GA techniques. The optimization target was set to the lowest possible Ra and the highest possible MRR and chip ratio.

## 3. Results and Discussion

### 3.1. Experimental Results

**Figure 3:**
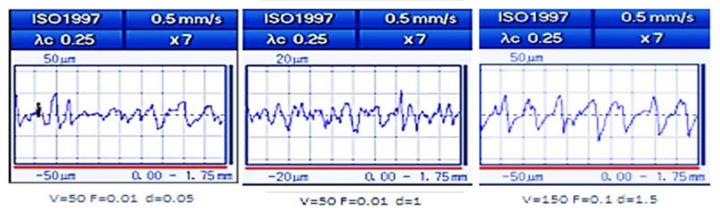
Surface profiles at different cutting conditions.

**Figure 4:**
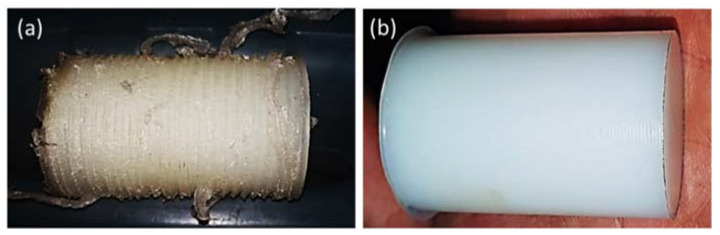
Photograph of the machined surface (**a**) worst surface, (**b**) best surface.

The surface characteristic of the machined part is affected by variations in the process input parameters (v_c_, f, and d). Figure 3 shows the roughness surface profiles at different cutting conditions. It is observed that Ra deteriorated at v_c_ max and f max. Smooth surfaces were obtained at low v_c_, f, and low-to-medium d. However, severe conditions that produced uneven surface finish were 150 mm/min, 0.1 mm/rev, and 1.5 mm, as shown in Figure 4a. Lower v_c_ of 50 mm/min, lower f of 0.01 mm/rev, and medium d of 1 mm was the combination that produced a smoother surface finish for PA6 of ≈2.4 µm, whereas d = 1.5 mm resulted in a smoother surface for HDPE of ≈2.12 µm, as shown in Figure 4b.

In addition to the surface roughness, the material removal rate also has an important role in the production of finished products. Higher MRR is preferred during machining in order to achieve high productivity, but with respect to the quality of the machined surface. As expected, it is obvious that, with an increase in v_c_, f, and, d, MRR increases as the contact area between the tool and workpiece increases. However, the roughness increases as MRR increases and chip area increases where: MRR = v_c_ × f × d mm^3^/min and chip area = f × d mm^2^.

**Figure 5:**
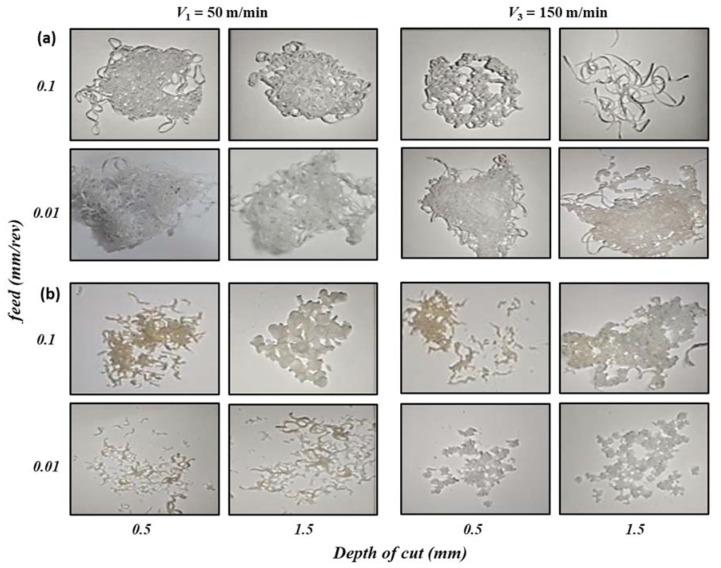
Optical images of chips obtained at different cutting conditions for (**a**) HDPE and (**b**) PA6.

The study of chip formation during machining operation has served a fundamental role to understand the mechanics of material removal. In addition, the determination of the chip thickness ratio is very important in the determination of material removal and surface roughness as it is one of the parameters that affect the shearing process and friction state at the entire chip/tool interface [23,33]. It is possible to recognize two main chip formation mechanisms: (I) continuous and (II) discontinuous chip. Based on these two types, the chips formed can be examined. Figure 5 shows the optical images of the collected chips formed at different v_c_, f, and d for (a) HDPE and (b) PA6. It was observed that low v_c_, f, and d, produce very long curly continuous chips, especially in case of HDPE machining. However, discontinuous chips are the common chip type formed during the machining of PA6. The transition between the two mechanisms is not sharp, as the chips appear, in some cases, highly stretched and with some melted filaments are observed. This behavior can be related to the fact that the machining of soft material produces helicoid continuous chips, as in the case of HDPE machining, which is more ductile and has relatively lower hardness compared to PA6. The high hardness of PA6 leads to discontinuous chip formation, which explains the higher values of surface roughness obtained in the case of PA6 compared to that of HDPE [34].

### 3.2. Statistical Results

#### 3.2.1. Regression Model

In this study, ANOVA was used to analyze the experimental data and determine the most significant impacts of input parameters (v_c_, f, and d) on the output responses (R_a_, MRR, and λc) by using the Design Expert software program (version 13.0.5, Stat-Ease, Inc., Minneapolis, MN, USA). Table 3 illustrates the summarized ANOVA results: F-value, *p*-value, lack of fit, adequate precision, R^2^, adjusted R^2^, and predicted R^2^ at a confidence level of 95%. The *p*-values of all responses were less than 0.05 and all F-values were greater than four, which implies the adequacy of the predicted models and that the independent parameters, as well as the individual model coefficients and interaction terms, had a significant effect on the responses. It was found that the most significant factor on Ra _HDPE_ was the depth of cut with an F-value of 86.4, in comparison to v_c_ with an F-value of 289.33 on Ra _PA6_. For both materials, HDPE and PA6, v_c_ had the greatest impact on MRR with F-values of 386.8 and 73.34, respectively. Finally, feed rate had a significant effect on λc for HDPE and PA6 with F-values of 2052 and 803.2, respectively.

The lack of fits for Ra and MRR for both materials, HDPE and PA6, were lower than 0.05, indicating that the model is insignificant. Chip thickness ratio (λc) had no lack of fit since the value of Sum of Squares (SS) of the regression model was larger than that of the residual. Moreover, the SS and mean squares (MS) of the pure error were equal to zero, so that there is no *p*-value and F-value for the lack of fit test. The signal to noise ratio (S/N) was computed with adequate precision to determine the model’s validity. It is recommended that S/N ratio exceed four [23]. The obtained responses’ adequate precision was greater than four and reveals that there was sufficient signal, and the model can be applicable to navigate the design space.

Many trails of regression transformation form and interaction between independent variables were examined in order to model the output responses. The relationship between the factors and the output parameters was modeled by quadratic regression. The best regression coefficient of determination (R^2^) indicates that the models generated in the experimental research are statistically significant when R^2^ is closer to 1 and can be used in predicting the response parameters with respect to the input control parameters [35].

**Figure 6:**
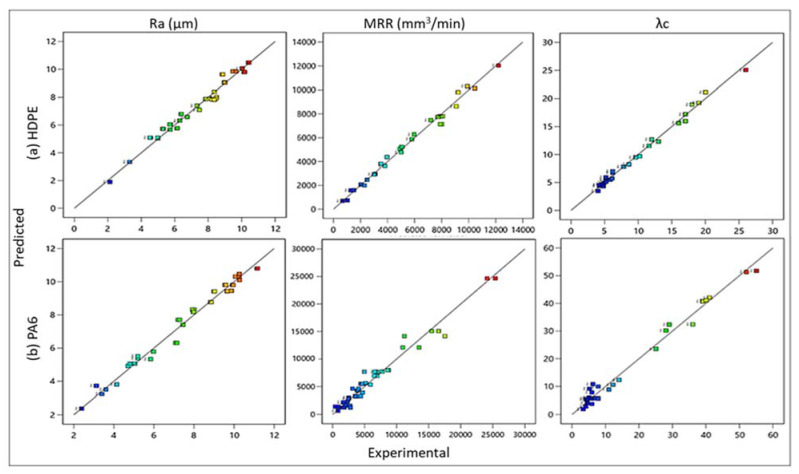
Comparison between experimental and predicted values of Ra, MRR, and λc for (**a**) HDPE and (**b**) PA6.

The regression Equations (1)–(3) represent the predicted non-linear model of HDPE responses and (4)–(6) of PA6 responses, Ra, MRR, and λc, as a function of v_c_, f, and d, with their associated determination and adjusted coefficients. In our study, R^2^ and adjusted R^2^ were very close to unity with values from 95.38–99.17%. Figure 6 illustrates the plot of the experimental values as a function of their corresponding predicted values of Ra, MRR, and λc for (a) HDPE and (b) PA6. By analyzing these figures, it became clear that there was a good agreement between experimental and predicted values, as most of the intersection points between them were very close to the median line, which confirms the effectiveness of the developed regression models.

Ra_HDPE_ = 4.56497 + 0.004158 v_c_ + 200.57640 f − 2.34411 d − 0.188974 v_c_ f + 0.140054 v_c_ d −107.44460 f d − 0.000067 v_c_^2^ − 1187.42747 f^2^ − 3.77600 d^2^ − 0.000615 v_c_^2^ d + 357.02546 f^2^ d + 38.98081 f d^2^ (*R*^2^ = 97.63%, *R*^2^*_adj_* = 96.93%) (1)

MRR_HDPE_ = 1564.63727 − 54.54011 v_c_ − 84010.80298 f − 583.17350 d + 877.35515 v_c_ f + 95.51891 v_c_ d + 60491.89891 f d + 0.326479 v_c_^2^ + 5.81515E+05 f ^2^ − 2269.31694 d^2^ − 619.97951 v_c_ f d + 3.36686 v_c_^2^ f − 0.544200 v_c_^2^ d − 6908.98148 v_c_ f ^2^ + 26.03333 v_c_ d^2^ + 94416.66667 f^2^ d + 4676.77596 f d^2^ (*R*^2^ = 99.10%, *R*^2^*_adj_* = 98.71%) (2)

λc _HDPE_ = −1.10886 + 0.355347 v_c_ − 165.02460 f + 4.09091 d − 3.42531 v_c_ f + 0.103812 v_c_ d − 155.10604 f d − 0.001872 v_c_^2^ +2627.13478 f^2^ + 0.025216 v_c_^2^ f −0.000514 v_c_^2^ d − 14.16798 v_c_ f ^2^ + 860.46391 f^2^ d (*R*^2^ = 99.17%, *R*^2^*_adj_* = 98.92%) (3)

Ra _PA6_ = 41.30189 − 0.807919 v_c_ + 357.63940 f − 85.42634 d − 2.44587 v_c_ f +1.78074 v_c_ d − 212.57032 f d + 0.003592 v_c_^2^ − 3679.04630 f^2^ + 39.09850 d^2^ − 0.283895 v_c_ f d + 0.007549 v_c_^2^ f − 0.007744 v_c_^2^ d + 52.69097 v_c_ f^2^ − 0.806673 v_c_ d^2^ + 1957.38194 f^2^ d + 39.52193 f d^2^ − 0.215384 v_c_^2^ f^2^ + 0.011114 v_c_^2^ f d + 0.003432 v_c_^2^ d^2^ − 17.83417 v_c_ f^2^ d (*R*^2^ = 98.46%, *R*^2^*_adj_* = 97.53%) (4)

MRR _PA6_ = −3991.74828 + 20.73222 v_c_ + 2.09063E+05 f +7175.40968 d −1312.86239 v_c_ f +14.49852 v_c_ d −3.30948E+05 f d − 1.15102E+06 f^2^ − 2551.52459 d^2^ + 1104.09016 v_c_ f d + 11503.14815 v_c_ f^2^ + 6.66509E+05 f^2^ d + 1.11560E+05 f d^2^ (*R*^2^ = 96.42%, *R*^2^*_adj_* = 95.38%) (5)

λc _PA6_ = 7.26518 + 0.462396 v_c_ − 216.08394 f + 29.83807 d − 6.64209 v_c_ f − 0.035717 v_c_ d − 740.25858 f d − 0.002598 v_c_^2^ + 2381.11133 f^2^ + 0.037648 v_c_^2^ f + 4966.21195 f^2^ d (*R*^2^ = 98.23%, *R*^2^*_adj_* = 97.82%) (6)

#### 3.2.2. Effect of Cutting Parameters on Ra

**Figure 7:**
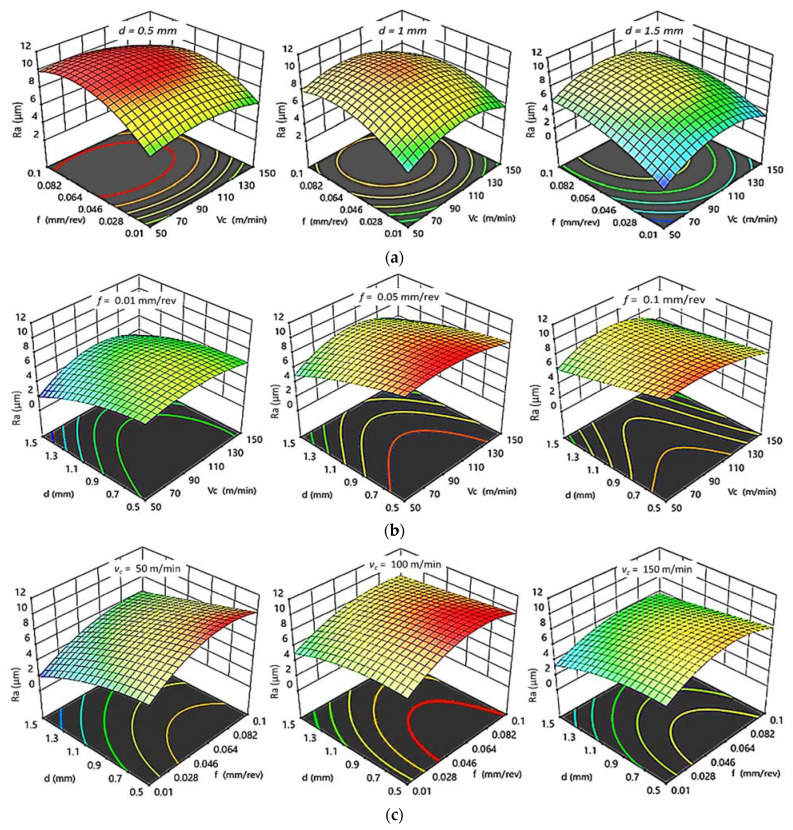
3D response plots for surface roughness of HDPE. (**a**) Effect of cutting speed and feed rate on Ra _HDPE_ at different depths of cut; (**b**) Effect of cutting speed and depth of cut on Ra _HDPE_ at different feed rates; (**c**) Effect of feed rate and depth of cut on Ra _HDPE_ at different cutting speeds.

**Figure 8:**
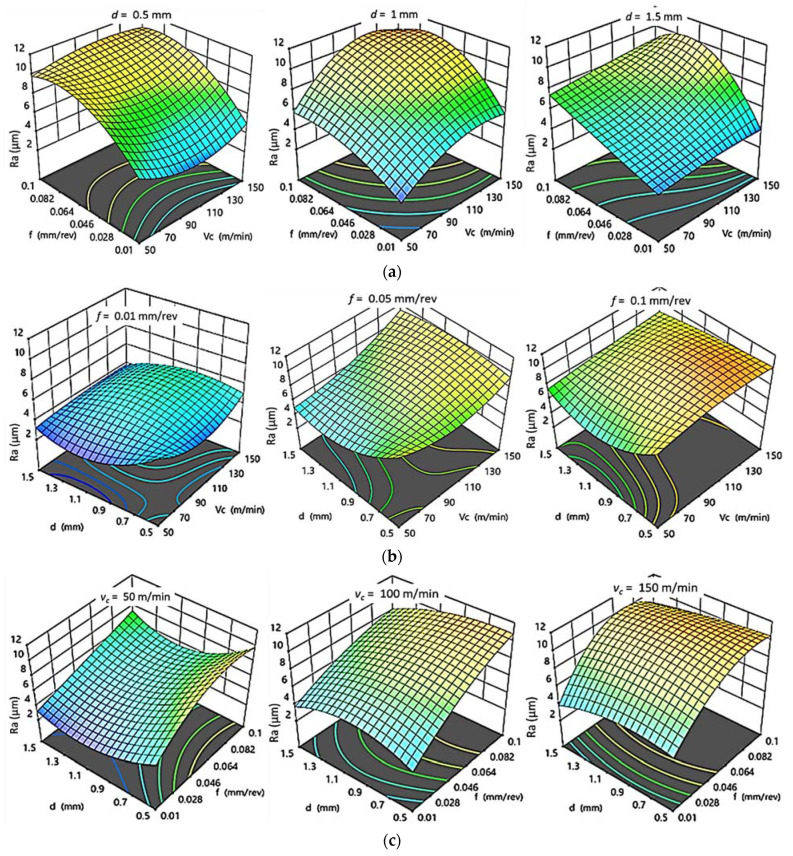
3D response plots for surface roughness of PA6. (**a**) Effect of cutting speed and feed rate on Ra _PA6_ at different depths of cut; (**b**) Effect of cutting speed and depth of cut on Ra _PA6_ at different feed rates; (**c**) Effect of feed rate and depth of cut on Ra _PA6_ at different cutting speeds.

Figure 7 and Figure 8 show 3D response plots that were constructed based on the regression models in order to evaluate the change in Ra as a function of cutting parameters (v_c_, *f*, and d) for HDPE and PA6, respectively.

For HDPE, as shown in Figure 7a, there is a proportional relationship between feed rate as well as cutting speed with Ra at constant depth of cut. It was observed that Ra increases rapidly with f and vc at certain depth of cut. At constant feed (Figure 7b), the increase in d produces better surface quality at low f; however, v_c_ does not show any significant changes in surface quality. Finally, it was concluded that Ra increases with increasing f and decreasing d at constant v_c_, and that the lower roughness is obtained at low v_c_ (Figure 7c). As a result, there is a fair agreement between interaction plots and experimental results. The minimal Ra is obtained at the lowest value of v_c_ and f and highest d, which agrees with the best Ra _HDPE_ ≈ 2.12 µm of experimental results obtained at 50 m/min, 0.01 mm/rev, and 1.5 mm.

For PA6, as shown in Figure 8a–c, it was concluded that the increase in both f and v_c_ result in increasing Ra while keeping d at constant level. However, by increasing d, Ra decreases until reaching its minimum value of 2.397 µm at 1 mm cutting depth. After that, Ra increases with further increasing of d. At constant v_c_, d has the same influence on Ra as at constant f, which has a proportional relation with roughness, whereas better surface finish is found at low v_c_. Accordingly, it is clear that the interaction plots results are compatible with the experimental results. The best surface finish is obtained at lowest v_c_, f, and 1 mm depth of cut, agreeing with the experimental results, which gave the best Ra _PA6_ ≈ 2.397 µm at 50 m/min, 0.01 mm/rev, and 1 mm.

#### 3.2.3. Effect of Cutting Parameters on MRR

**Figure 9:**
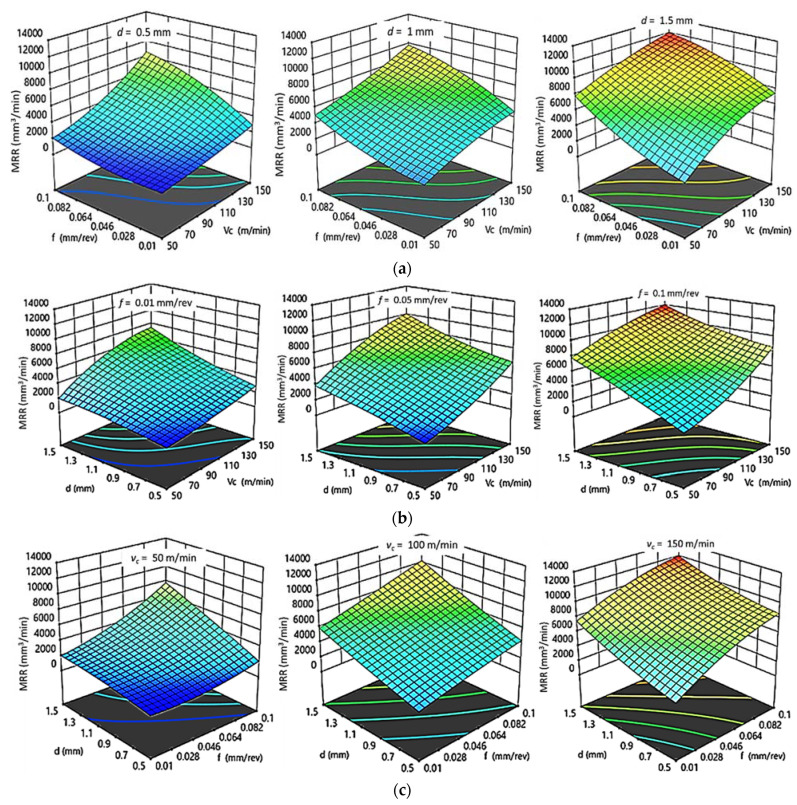
3D response plots for material removal rate of HDPE. (**a**) Effect of cutting speed and feed rate on MRR _HDPE_ at different depths of cut; (**b**) Effect of cutting speed and depth of cut on MRR _HDPE_ at different feed rates; (**c**) Effect of feed rate and depth of cut on MRR _HDPE_ at different cutting speeds.

**Figure 10:**
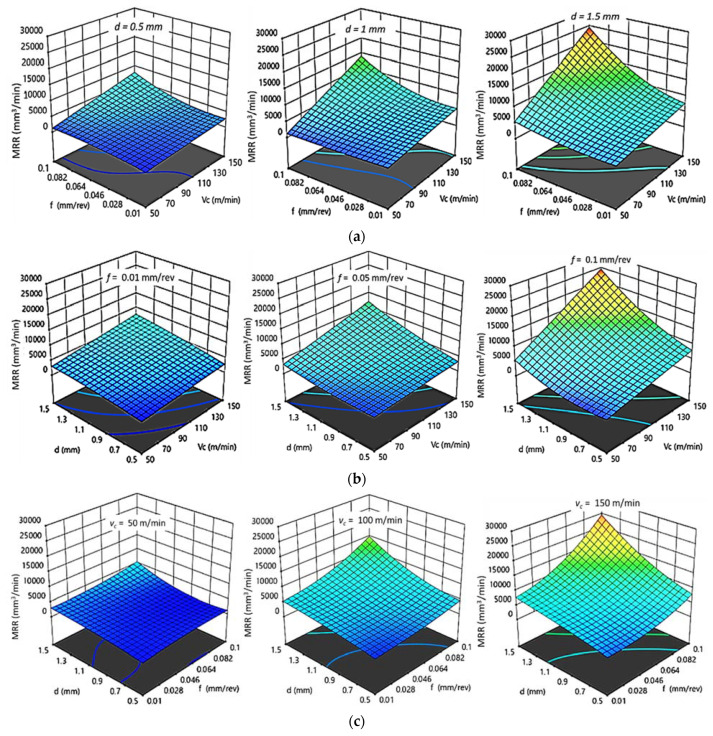
3D response plots for material removal rate of PA6. (**a**) Effect of cutting speed and feed rate on MRR _PA6_ at different depths of cut; (**b**) Effect of cutting speed and depth of cut on MRR _PA6_ at different feed rates; (**c**) Effect of feed rate and depth of cut on MRR _PA6_ at different cutting speeds.

The effects of cutting parameters on MRR for HDPE and PA6 obtained by regression models are shown in Figure 9 and Figure 10, respectively. It was noticed that there was a similar effect of cutting parameters on MRR for both HDPE and PA6. MRR increased with increases in v_c_, f, and d. There is agreement between interaction plots and experimental results. The best MRRs of HDPE and PA6 obtained from interaction plots were found at high v_c_, f, and d, which agrees with the experimental results, as best MRR _HDPE_ = 12,200 mm^3^/min and MRR _PA6_ = 25,394 mm^3^/min were obtained at 150 m/min, 0.1mm/rev, and 1.5 mm. In addition, it was noted that MRR _HDPE_ is half MRR _PA6_.

#### 3.2.4. Effect of Cutting Parameters on λc

**Figure 11:**
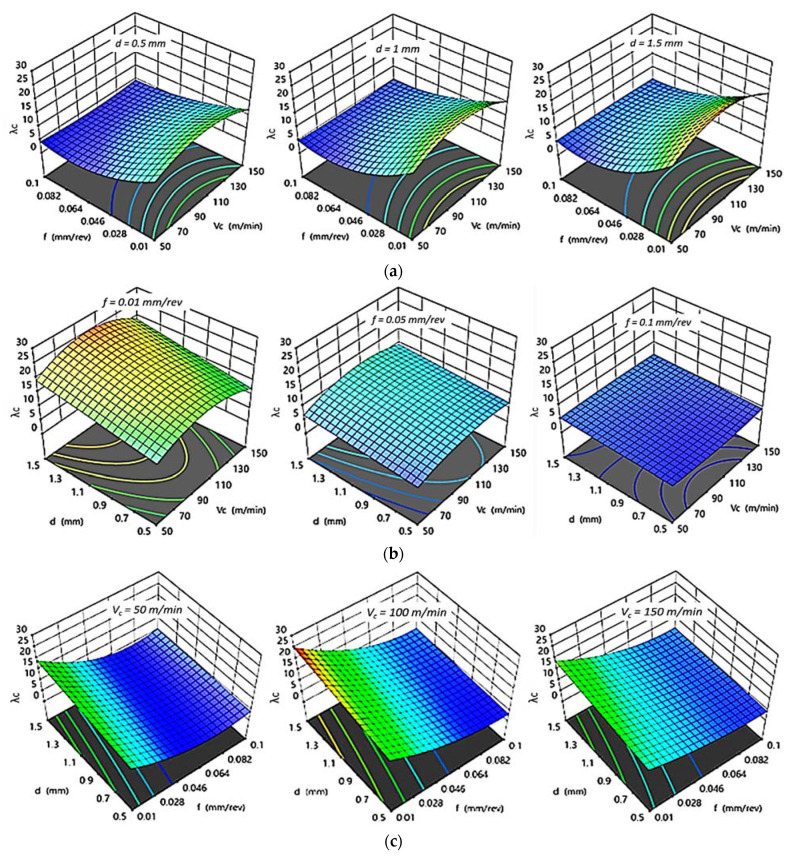
3D response plots for chip ratio of HDPE. (**a**) Effect of cutting speed and feed rate on λc _HDPE_ at different depths of cut; (**b**) Effect of cutting speed and depth of cut on λc _HDPE_ at different feed rates; (**c**) Effect of feed rate and depth of cut on λc _HDPE_ at different cutting speeds.

**Figure 12:**
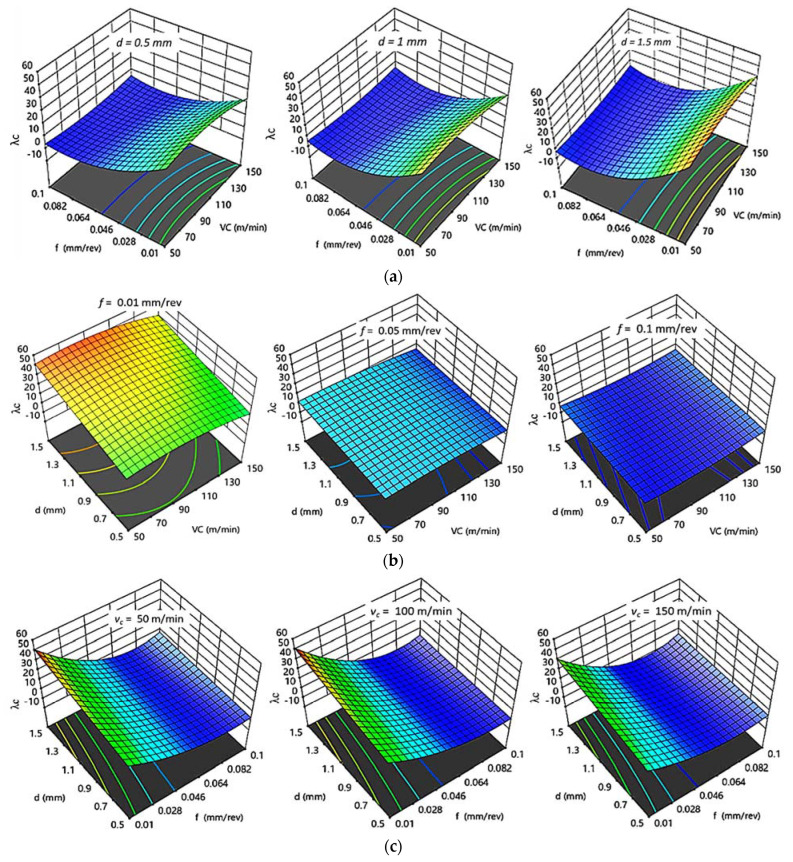
3D response plots for chip ratio of PA6. (**a**) Effect of cutting speed and feed rate on λc _PA6_ at different depths of cut; (**b**) Effect of cutting speed and depth of cut on λc _PA6_ at different feed rates; (**c**) Effect of feed rate and depth of cut on λc _PA6_ at different cutting speeds.

Figure 11 and Figure 12 illustrate the influence of changing cutting parameters obtained by regression models on the chip ratios (λc) for HDPE and PA6, respectively. The same behavior of chip ratio was remarked for both HDPE and PA6. At constant d, an increasing of f decreased the λc while an increasing of v_c_ rose the λc to its optimum value at 100 m/min; then, λc decreased again with a further increasing of v_c_. The chip ratio also increased by an increasing of d, even at a fixed level of f or v_c_. The higher the chip thickness ratio attained, the better the surface finish was. A higher λc for both HDPE and PA6, with values of 26.036 and 55.077, respectively, was achieved at v_c_ of 100 m/min, lower f of 0.01 mm/rev, and highest d of 1.5 mm, which confirms the experimental with the interaction plots results.

### 3.3. Optimization Results

#### 3.3.1. RSM Results

**Figure 13:**
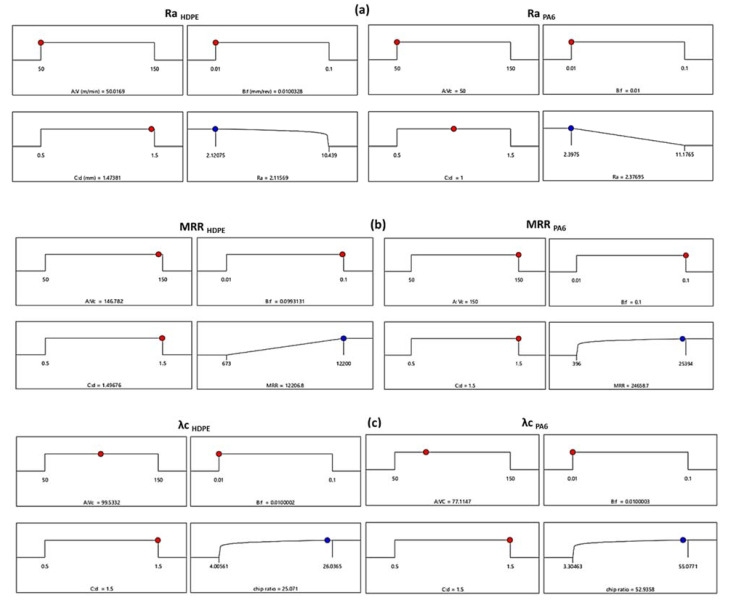
RSM optimization results of (**a**) Ra, (**b**) MRR, and (**c**) λc for HDPE and PA6.

Based on the comprehensive analysis of each independent variable, the process optimization was developed to obtain the best parameter combination for HDPE and PA6 turning for the desire responses using the Design Expert software (version 13.0.5, Stat-Ease, Inc., Minneapolis, MN, USA). Figure 13a shows the optimized surface roughness (Ra) of turning HDPE and PA6 and corresponding conditions using the desirability function of the RSM. For all the following optimization findings the red dot and the blue dotes the indicated the cutting condition (v_c_, f and d) and response (Ra, MRR and λc) respectively. As the machinability is better when Ra is low, so the solution destination was set to “Minimize”, the optimization target was set to “In range”, and the desirability function’s predicted output was in the form of “smaller-is-better”. For HDPE, the minimum surface roughness value of 2.1156 µm was predicted using the combination of v_c_ (A) = 50 m/min, f (B) = 0.01 mm/rev, and d (C) = 1.47 mm. For PA6, the minimum Ra _PA6_ value of 2.376 µm was predicted using the combination of v_c_ (A) = 50 m/min, f (B) = 0.01 mm/rev, and d (C) = 1 mm.

Figure 13b presents the RSM optimization results of MRR and corresponding conditions. The optimization target was set to “In range”, the solution destination was set to “Maximize”, and the desirability function’s predicted output was in the form of “larger-is-better”. For HDPE, the optimal cutting conditions values were v_c_ (A) = 146.782 m/min, f (B) = 0.099 mm/rev, and d (C) = 1.496 mm for a maximum MRR _HDPE_ value of 12206.8 mm^3^/min. For PA6, the optimal cutting conditions values were v_c_ (A) = 150 m/min, f (B) = 0.1 mm/rev, and d (C) = 1.5 mm for a maximum MRR _PA6_ value of 24,658.7 mm^3^/min.

The RSM optimization results of the chip ratio (λc) and their corresponding conditions are shown in Figure 13c. As the higher chip ratio obtained, the better surface finish is, so that the optimization target was set to “In range”, the solution destination was set to “Maximize”, and the desirability function’s predicted output was in the form of “larger-is-better” characteristics. For HDPE, a maximum chip ratio value of 25.071 was attained at optimum cutting condition values of v_c_ (A) = 99.533 m/min, f (B) = 0.01 mm/rev, and d (C) = 1.5 mm. For PA6, the optimum cutting condition combination values were v_c_ (A) = 77.11 m/min, f (B) = 0.01 mm/rev, and d (C) = 1.5 mm for a maximum chip ratio value of 52.935.

For a confirmation test, the optimum cutting conditions and responses acquired by RSM are compared to the GA and hybrid FFD-GA results developed in the next section.

#### 3.3.2. GA and Hybrid FFD-GA Results

A genetic algorithm (GA) was used to find out the optimum set of cutting independent variables that contribute to the lowest possible Ra and the highest possible MRR and λc. Equations (1)–(6) for each response were taken as the objective function and subjected to the cutting boundary conditions, v_c_, f, and d, by using a genetic algorithm approach. Proposed objective functions can be expressed as follows:

Minimize (v_c_, f, d)

Subjected to ranges of cutting conditions:

50 ≤ v_c_ ≤ 150 (m/min), 0.01≤ f ≤ 0.1 (mm/rev), 0.5 ≤ d ≤ 1.5 (mm)

**Figure 14:**
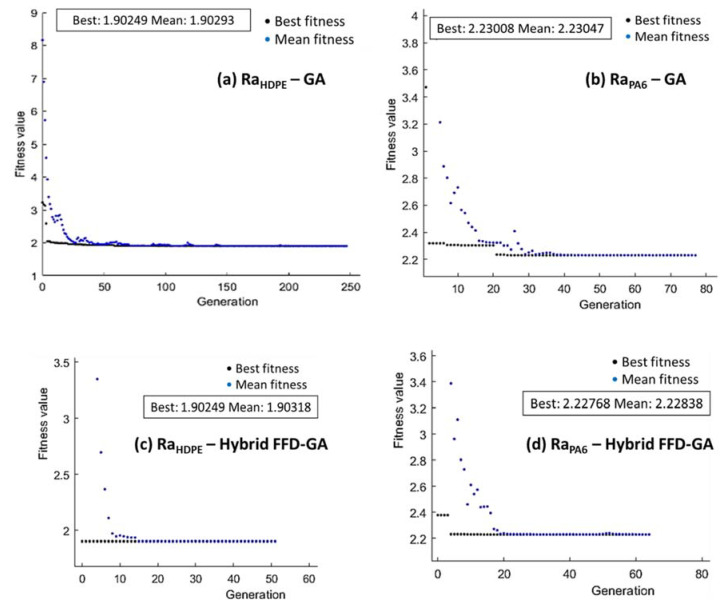
Optimum Ra of HDPE (**a**,**c**) and PA6 (**b**,**d**) by GA (**a**,**b**) and hybrid FFD-GA (**c**,**d**).

**Figure 15:**
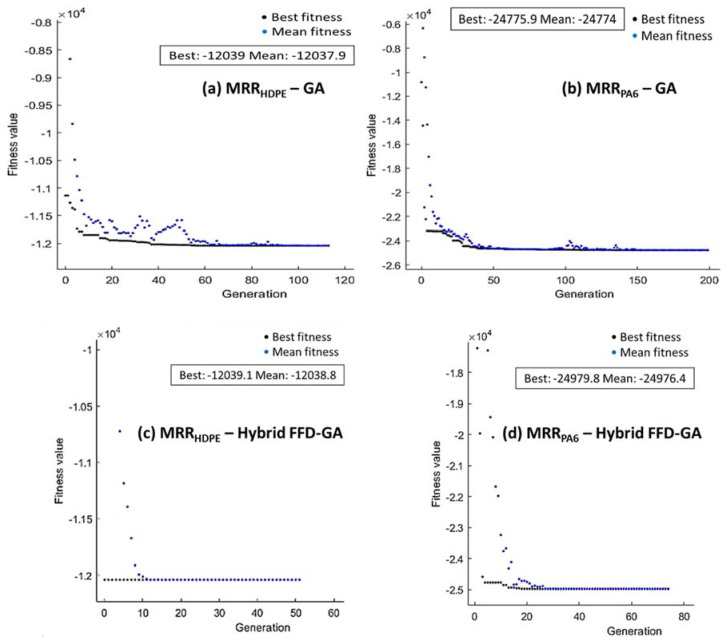
Optimum MRR of HDPE (**a**,**c**) and PA6 (**b**,**d**) by GA (**a**,**b**) and hybrid FFD-GA (**c**,**d**).

**Figure 16:**
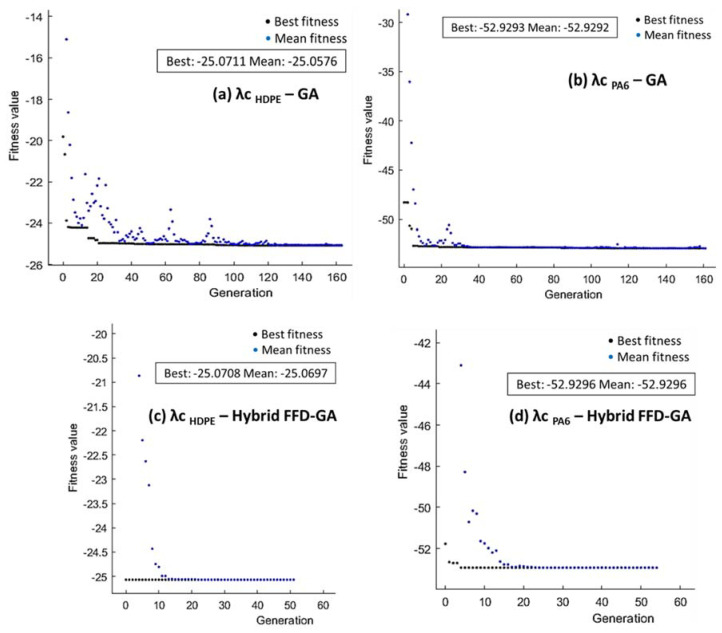
Optimum λc of HDPE (**a**,**c**) and PA6 (**b**,**d**) by GA (**a**,**b**) and hybrid FFD-GA (**c**,**d**).

For the GA optimization technique, Figure 14, Figure 15 and Figure 16 present the performance of fitness value and run solver view generated from MATLAB and corresponding cutting conditions of the best Ra, MRR, and λc, respectively, for HDPE and PA6.

The minimization of Ra proposed in Equations (1) and (4) was taken as the fitness function and subjected to the cutting boundary condition. The minimum value of Ra _HDPE_ obtained by GA was 1.902 µm at v_c_ = 50 m/min, f = 0.01 mm/rev, and d = 1.5 mm (Figure 14a). In addition, the minimum value of Ra _PA6_ was 2.23 µm, obtained by GA at v_c_ = 50 m/min, f = 0.01 mm/rev, and d = 1.139 mm (Figure 14b).

In order to improve the results generated from GA, a hybrid of full factorial design and GA (FFD-GA) was performed. Initial populations of hybrid FFD-GA based on FFD optimum cutting conditions were: v_c_ = 50 m/min, f = 0.01 mm/rev, and d = 1.5 mm, and v_c_ = 50 m/min, f = 0.01 mm/rev, and d = 1.5 mm for HDPE and PA6, respectively. The minimum Ra value for HDPE (Figure 14c) obtained by hybrid FFD-GA was 1.902 µm at 50 m/min, 0.01 mm/rev, and 1.5 mm. For PA6, the minimum Ra obtained by hybrid FFD-GA was 2.227 µm (Figure 14d) at 50 m/min, 0.01 mm/rev, and 1.139 mm.

The maximization of MRR proposed in Equations (2) and (5) was taken as the fitness function and subjected to the cutting boundary condition. The best values of MRR_HDPE_ and MRR_PA6_ by GA, shown in Figure 15a,b, were 12,039 mm^3^/min and 24,775.9 mm^3^/min, respectively, obtained at 150 m/min, 0.1 mm/rev, and 1.5 mm. Hybrid FFD-GA results of maximum MRR _HDPE_ and MRR _PA6_ were 12,039.1 mm^3^/min and 24,979.8 mm^3^/min, respectively, obtained at 150 m/min, 0.1 mm/rev, and 1.5 mm, as shown in Figure 15c,d.

The maximization of λc proposed in Equation (3) for HDPE and Equation (6) for PA6 were taken as the fitness function and subjected to the cutting boundary condition. As shown in Figure 16a,b, GA results indicated that the best value of λc _HDPE_ was 25.0711 obtained at 99.4 m/min, 0.01 mm/rev, and 1.5 mm whereas λc _PA6_ was 52.9293 obtained at 75.735 m/min, 0.01 mm/rev, and 1.5 mm. Hybrid FFD-GA results shown in Figure 16 c,d state that the maximum λc _HDPE_ = 25.07 was obtained at 99 m/min, 0.01 mm/rev, and 1.5 mm. In addition, the maximum value of λc_PA6_ = 52.92 was obtained at 75.665 m/min, 0.01 mm/rev, and 1.5 mm.

#### 3.3.3. Multi-Objective Genetic Algorithm Optimization

The multi-objective genetic algorithm (MOGA) methodology was used to solve a mathematical model in which the input process parameters affects the output responses quality [36]. In the current study, multi-objective optimization using genetic algorithm (MOGA) has been used as the objective function in MATLAB 2020’s GA Toolbox. The objective functions, fitness functions, are essential in GA to solve optimization problems and regression models were used to be the fitness function of the current optimization problem. The upper and lower boundaries were set based on turning input parameter values (v_c_, f, d) and the number of variables was set to three. The MOGA parameters selected were as follows: initial population size was 50, optimization was achieved by setting intermediate crossover with a probability of 0.8 and constraint dependent mutation, the generation size was 300, the migration interval was 20, the migration fraction was 0.2, and the Pareto fraction was 0.35. The result of MOGA is the Pareto optimum, a non-dominated solution, which is a set of solutions that take into account all of the objectives while not losing any of them [37].

##### MOGA of Ra and MRR

**Figure 17:**
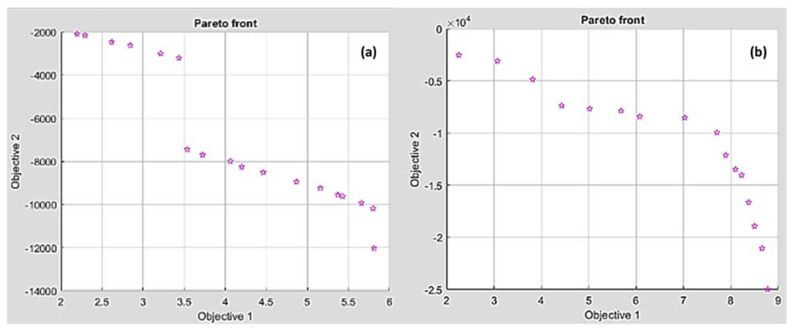
Pareto front chart of Ra and MRR for (**a**) HDPE and (**b**) PA6.

The genetic algorithm, a non-traditional optimization technique, was used to utilize the minimum of Ra and maximum of MRR by considering both as multi-objective functions. For HDPE, the fitness functions of Ra and MRR were Equations (1) and (2), respectively. For PA6, the fitness functions of Ra and MRR were Equations (4) and (5), respectively. Table 4 lists the Pareto front points of Ra and MRR for HDPE and PA6 obtained by MOGA. Figure 17 presents the Pareto chart points of Ra (Objective 1) and MRR (Objective 2) for HDPE and PA6. It was noticed that high MRR produces rough surface. Consequently, the best surface finish can be achieved by sacrificing an increase in MRR. For HDPE, the best Ra (2.19 µm) had a minimal MRR of 2085 mm^3^/min and the best MRR (12,024 mm^3^/min) had the worst Ra with a value of 5.8 µm. For PA6, the best Ra (2.25 µm) had a minimal MRR of 2523 mm^3^/min and the best MRR (24,967.4 mm^3^/min) had the worst Ra with a value of 8.77 µm.

##### MOGA of Ra and λc

**Figure 18:**
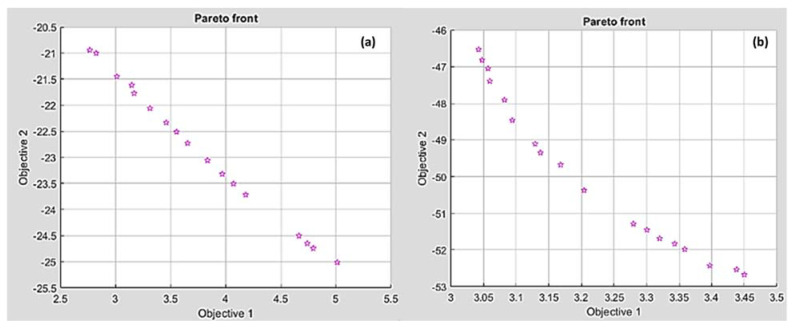
Pareto front chart of Ra and chip ratio for (**a**) HDPE and (**b**) PA6.

Minimal Ra and maximum λc were used as multi-objective functions using a genetic algorithm. Equations (1) and (3) were the fitness functions of Ra and λc for HDPE, respectively. Equations (4) and (6) were used to calculate the fitness functions of Ra and λc for PA6. Pareto front points of Ra and λc for HDPE and PA6 are listed in Table 5. Figure 18 shows Pareto chart points of Ra (Objective 1) and λc (Objective 2) for (a) HDPE and (b) PA6. It was revealed that the increase in chip ratio produces a rough surface. Therefore, the surface finish can be improved with the sacrifice of an increase in chip ratio. For HDPE, the best Ra (2.76 µm) had a minimal λc of 20.94 and the best chip ratio (25.01) had the worst Ra with a value of 5.01 µm. For PA6, the best Ra (3.04 µm) had a minimal λc of 46.5 and the best chip ratio (52.67) had the worst Ra with a value of 3.45 µm.

## 4. Conclusions

This study dealt with experimental investigations on some aspects of machinability such as surface roughness, material removal rate, and chip formation during the turning of two common types of polymers: HDPE and PA6, difficult-to-cut materials, using a cemented carbide insert (K10) tool.

Based on the experimental results, statistical analysis tools such as ANOVA and 3D response surface plots were evaluated. Moreover, RSM, GA, hybrid FFD-GA, and MOGA were developed to determine the optimal cutting parameters’ setting, minimizing surface roughness and maximizing the material removal rate and chip thickness ratio. The following conclusions were drawn:

The most significant factor on Ra _HDPE_ is the depth of cut whereas cutting speed is most significant on Ra_PA6_. For both materials, HDPE and PA6, cutting speed has the greatest impact on MRR and feed rate has a significant effect on λc for HDPE and PA6.

Ra increases with feed rate and cutting speed; however, the increase of depth of cut produces better surface quality. A high MRR of HDPE is found at high cutting speed, feed rate, and depth of cut, which affects the surface finish negatively. Better surface finish can be achieved at higher chip thickness ratio, which is attained by decreasing the feed rate while increasing both cutting speed and depth of cut.

Satisfactory correlations were acquired between experimental and predicted results for surface roughness criteria, material removal rate, and chip ratio.

The comparison of turning responses’ values of experimental results obtained by FFD, optimization results by RSM, optimization results by GA, hybrid FFD-GA, and MOGA for HDPE and PA6 is summarized in Table 6.

## Figures and Tables

**Table 1:** Main properties of tested polymers.

Material	Physical Properties	Mechanical Properties		Thermal Properties	
	Density	HB	Tensile Strength	Thermal Conductivity	Melting Temperature
	(gm/cm^3^)	(MPa)	(Mpa)	(W/km)	(°C)
HDPE	0.95~0.98	48.3	21	0.396	221
PA6	1.14	150	76	0.25	340

**Table 2:** Full factorial design of experiment and the response results.

Test Order	Machining Parameters			Response Variables					
	vc (m/min)	f (mm/rev)	d (mm)	HDPE			PA6		
				Ra (µm)	MRR (mm^3^/min)	λc	Ra (µm)	MRR (mm^3^/min)	λc
1	50	0.01	1	4.528	1300	17.0238	2.3975	1700	39.0546
2	100	0.05	1	8.8405	5000	10.2143	8.0205	3845	7.81093
3	150	0.05	1.5	5.27075	10,450	9.6134	8.99725	10,962	7.81093
4	50	0.05	0.5	10.03475	1030	4.8067	9.88	834	11.0154
5	100	0.1	0.5	10.1365	3961	4.5056	11.14475	4046	3.3046
6	50	0.01	1.5	2.12075	2300	19.0266	3.4005	2543	52.0729
7	50	0.05	1.5	4.98275	3500	6.208	4.91625	3587	14.0196
8	150	0.1	1.5	6.1325	12,200	5.5085	8.8235	25,394	5.8081
9	150	0.1	1	6.37425	9200	5.2072	9.91925	17,525	4.5063
10	150	0.1	0.5	7.845	9080	4.2058	10.2665	8750	4.1057
11	50	0.01	1	4.528	1300	16.0238	2.3975	1700	39.0546
12	50	0.05	1	8.49575	2480	5.2072	4.70925	2317	12.2171
13	150	0.05	1	8.38075	7720	8.61205	10.271	6975	7.8109
14	150	0.01	1.5	3.29975	7200	17.0252	4.144	7731	40.05607
15	100	0.01	1.5	4.981	5800	25.0364	3.1245	5900	55.0771
16	100	0.05	1	8.87125	5090	11.2143	7.923	3910	7.8109
17	100	0.1	1	8.958	7900	4.6064	10.256	6604	4.1057
18	100	0.1	0.5	10.179	3960	4.0056	11.1765	3125	3.3046
19	50	0.01	0.5	6.7405	673	11.0168	5.203	850	28.0392
20	50	0.1	1.5	5.7525	7932	5.8081	7.4535	4383	5.9082
21	50	0.1	0.5	9.476	2019	4.2058	9.595	1842	4.5063
22	50	0.1	1	8.48575	4904	4.9068	5.97225	2769	5.1071
23	100	0.01	1	7.47	3800	19.02803	5.8395	4680	41.0574
24	100	0.01	1.5	4.981	5800	26.0364	3.1245	5900	55.0771
25	50	0.1	0.5	9.6645	2020	5.2058	9.5435	1547	4.5063
26	50	0.1	1	8.08575	4952	4.6068	5.98725	2769	5.1071
27	100	0.01	1	7.47	3800	20.02803	5.8395	4680	41.0574
28	50	0.01	0.5	6.7405	673	12.0168	5.203	850	28.0392
29	50	0.01	1.5	2.12075	2300	18.2266	3.4005	2543	52.0729
30	150	0.05	1.5	5.32625	10,450	8.9134	9.02825	13,487	7.8109
31	150	0.01	1	5.7195	5000	16.0224	5.2	5150	29.0406
32	150	0.05	0.5	8.9865	5970	6.2086	9.66425	3506	6.8095
33	150	0.1	1	6.394	9230	5.7072	9.962	11,210	4.5063
34	150	0.1	1.5	6.15825	12,200	6.1085	8.87075	24,125	5.8081
35	100	0.1	1	9.0045	8020	4.9064	10.06275	4962	4.1057
36	100	0.05	1.5	8.20875	7930	11.6162	7.1545	6921	6.2086
37	50	0.1	1.5	5.70625	8088	6.3081	7.452	4641	5.9082
38	150	0.1	0.5	7.8625	9080	4.8058	10.26575	8578	4.1057
39	150	0.01	0.5	6.30775	3000	12.0182	5.0435	2500	25.03504
40	150	0.05	1	8.362	7730	8.11205	10.2755	6500	7.81093
41	50	0.05	0.5	10.03475	1030	5.4067	9.86975	396	11.0154
42	100	0.01	0.5	7.333	1500	16.0238	3.60275	1980	36.0504
43	100	0.1	1.5	8.3735	9900	5.2072	7.987	16,547	5.9082
44	50	0.05	1.5	4.98275	3517	6.0086	4.79075	3813	14.0196
45	150	0.05	0.5	8.9885	5970	6.8086	9.647	4375	6.8095
46	100	0.01	0.5	7.333	1500	17.0238	3.60275	1980	36.0504
47	100	0.05	1.5	8.2185	7800	10.6162	7.0805	6921	6.2086
48	150	0.01	1.5	3.29975	7200	18.0252	4.144	7731	40.05607
49	150	0.01	1	5.7195	5000	16.4224	5.2	5150	29.0406
50	50	0.05	1	8.49575	2490	5.00729	4.71075	2728	12.2171
51	150	0.01	0.5	6.30775	3000	13.0182	5.0435	2500	25.03504
52	100	0.1	1.5	8.3675	9900	5.70729	7.9625	15,470	5.9082
53	100	0.05	0.5	10.4005	3090	8.31093	7.20675	2348	5.20729
54	100	0.05	0.5	10.439	3060	7.8109	7.2955	2327	5.20729

**Table 3:** ANOVA summarized results of cutting responses.

	Response	F-Value (F > 4)	*p*-Value (*p* < 0.05)	Lack of Fit (*p* > 0.05)	Adeq Precision (ratio > 4)	R^2^	R^2^_adj_	R^2^_pred_
HDPE	Ra	140.44	<0.0001	0.0001	47.3369	0.9763	0.9693	0.9623
	MRR	254.84	<0.0001	0.0001	55.6343	0.991	0.9871	0.9809
	λc	407.08	<0.0001	----	69.8882	0.9917	0.9892	0.9853
PA6	Ra	105.8	<0.0001	0.0001	32.9903	0.9846	0.9753	0.9601
	MRR	92.08	<0.0001	0.0001	41.9425	0.9642	0.9538	0.9397
	λc	238.39	<0.0001	----	46.0162	0.9823	0.9782	0.9725

**Table 4:** Paterian points of Ra and MRR for HDPE and PA6.

No.	HDPE					PA6				
	Conditions			Responses		Conditions			Responses	
	v_c_	f	d	Ra	MRR	v_c_	f	d	Ra	MRR
1	50.10064	0.01263	1.49849	2.19748	−2085.9045	149.96604	0.09998	1.4999	8.77499	−24967.431
2	149.84927	0.09970	1.49925	5.80914	−12024.143	50.01438	0.01039	1.1419	2.25688	−2523.2370
3	149.57741	0.04108	1.49905	5.42572	−9610.6830	143.79544	0.016179	1.4831	5.01879	−7684.1109
4	149.57899	0.04663	1.49907	5.65631	−9929.6614	149.96604	0.09998	1.4999	8.77499	−24967.431
5	53.91927	0.01265	1.49807	2.61807	−2463.5907	106.11727	0.08151	1.4992	7.88962	−12127.141
6	61.51459	0.01341	1.49789	3.43465	−3201.2878	123.34297	0.09935	1.4989	8.49748	−18926.685
7	149.69267	0.05095	1.49899	5.798399	−10168.033	133.90288	0.09894	1.4990	8.6559	−21049.551
8	149.55036	0.02395	1.49895	4.462317	−8504.0965	91.28852	0.011075	1.4863	3.81448	−4866.6248
9	149.57155	0.030339	1.49898	4.865125	−8937.9101	97.71378	0.09749	1.4975	8.09410	−13485.296
10	148.61716	0.01027	1.49206	3.53617	−7438.4793	145.99145	0.011315	1.4382	4.42666	−7376.4625
11	50.67217	0.01287	1.4974	2.29414	−2150.3586	141.42424	0.02086	1.4867	5.68027	−7869.9244
12	59.24755	0.01333	1.49849	3.21338	−2992.0239	102.11577	0.09706	1.4873	8.22486	−14029.913
13	149.75849	0.02045	1.49892	4.19981	−8259.0439	113.96231	0.04970	1.4930	7.02449	−8528.3537
14	149.35865	0.03504	1.49762	5.15621	−9227.7809	103.23314	0.07284	1.4666	7.70323	−9950.7383
15	148.68008	0.01715	1.49639	4.0611	−7985.3721	114.12946	0.09763	1.4988	8.37259	−16651.477
16	55.16604	0.01355	1.49703	2.84483	−2612.9313	148.09737	0.02223	1.4918	6.07418	−8419.7041
17	148.95875	0.01319	1.49636	3.72502	−7693.2979	57.51669	0.01194	1.3257	3.07088	−3099.2571
18	149.58656	0.03989	1.49899	5.37042	−9539.5601	123.34297	0.09935	1.4989	8.49748	−18926.685

**Table 5:** Paterian points of R_a_ and λc for HDPE and PA6.

No.	HDPE					PA6				
	Conditions			Responses		Conditions			Responses	
	v_c_	f	d	Ra	λc	v_c_	f	d	Ra	λc
1	95.207315	0.010036	1.49981	5.011362	−25.011145	59.786200	0.010018	1.2251	3.04235	−46.53234
2	58.077290	0.010086	1.49961	2.765689	−20.945216	62.429872	0.010020	1.4997	3.39714	−52.42544
3	75.848055	0.010051	1.49959	4.180225	−23.717750	60.902873	0.010053	1.4783	3.34333	−51.83081
4	65.792705	0.010068	1.49921	3.459049	−22.332439	59.823397	0.010032	1.4107	3.20404	−50.37390
5	66.964083	0.010090	1.49970	3.552603	−22.511376	59.804024	0.010025	1.2395	3.04768	−46.82421
6	86.659306	0.010063	1.49946	4.739315	−24.649591	59.894496	0.010062	1.2933	3.08193	−47.90476
7	95.207315	0.010036	1.49981	5.011362	−25.011145	60.299039	0.010035	1.4611	3.30060	−51.45451
8	68.287165	0.010051	1.49958	3.653390	−22.729087	59.911380	0.010085	1.3803	3.16817	−49.68074
9	62.426334	0.010067	1.49972	3.167762	−21.768498	59.830667	0.010022	1.3606	3.13724	−49.34672
10	88.119805	0.010047	1.49973	4.793607	−24.743113	59.815306	0.010025	1.3183	3.09407	−48.46213
11	64.085893	0.010063	1.49973	3.311835	−22.057325	60.990353	0.010066	1.4862	3.35857	−51.98181
12	74.097835	0.010067	1.49970	4.069230	−23.506688	59.816572	0.010024	1.2670	3.05946	−47.39907
13	60.690432	0.010069	1.49968	3.011109	−21.453352	59.883487	0.010025	1.2501	3.05674	−47.05214
14	61.994241	0.010225	1.49958	3.146351	−21.619485	60.417895	0.010023	1.4711	3.32010	−51.68791
15	58.576330	0.010174	1.49958	2.823226	−21.003742	66.016942	0.010023	1.4953	3.43826	−52.53416
16	70.669659	0.010076	1.49965	3.833709	−23.058982	59.921783	0.010026	1.3488	3.12919	−49.10542
17	72.587857	0.010062	1.49967	3.967493	−23.320492	60.017329	0.010023	1.4536	3.27977	−51.29148
18	84.806305	0.010121	1.49975	4.664187	−24.504420	66.986410	0.010023	1.4999	3.45013	−52.67488

**Table 6:** Summary results of HDPE and PA6 by turning process.

HDPE	**Response**		**FFD**	**RSM**	**GA**	**FFD-GA**	**MOGA**	
							**Ra, MRR**	**Ra, λc**
	Ra	Value	2.12075	2.11569	1.90249	1.90249	2.197	2.76
		v_c_	50	50.0169	50	50	50.1	58.07
		f	0.01	0.0100328	0.01	0.01	0.012	0.01
		d	1.5	1.47381	1.5	1.5	1.498	1.49
	MRR	Value	12,200	12,206.8	12,039	12,039.1	12,024.143	
		v_c_	150	146.782	150	150	149.84	
		f	0.1	0.0993131	0.1	0.1	0.099	
		d	1.5	1.49676	1.5	1.5	1.49	
	λc	Value	26.0365	25.071	25.0711	25.0708		25.01
		v_c_	100	99.5332	99.425	99.032		95.2
		f	0.01	0.01	0.01	0.01		0.01
		d	1.5	1.5	1.5	1.5		1.499
PA6	Ra	Value	2.3975	2.3769	2.23008	2.22768	2.25	3.042
		v_c_	50	50	50	50	50.01	59.78
		f	0.01	0.01	0.01	0.01	0.0103	0.01
		d	1	1	1.139	1.139	1.149	1.225
	MRR	Value	25,394	24,658.7	24,775.9	24,979.8	24,967.431	
		v_c_	150	150	149.113	150	149.96	
		f	0.1	0.1	0.1	0.1	0.099	
		d	1.5	1.5	1.5	1.5	1.49	
	λc	Value	55.0771	52.9358	52.9293	52.9296		52.67
		v_c_	100	77.1147	75.735	75.665		66.98
		f	0.01	0.01	0.01	0.01		0.01
		d	1.5	1.5	1.5	1.5		1.49

## Data Availability

All the raw data supporting the conclusion of this paper were provided by the authors.

## References

[1] Bozdemir M. (2017). The Effects of Humidity on Cast PA6G during Turning and Milling Machining. Adv. Mater. Sci. Eng..

[2] Palanikumar K., Rajasekaran T., Latha B. (2015). Fuzzy rule-based modeling of machining parameters for surface roughness in turning carbon particle-reinforced polyamide. J. Thermoplast. Compos. Mater..

[3] Patel P., Chaudhary V., Patel K., Gohil P. (2018). Milling of Polymer Matrix Composites: A Review. Int. J. Appl. Eng. Res..

[4] Dehghan Manshadi M., Alafchi N., Tat A., Mousavi M., Mosavi A. (2022). Comparative Analysis of Machine Learning and Numerical Modeling for Combined Heat Transfer in Polymethylmethacrylate. Polymers.

[5] Gnatowski A., Gołębski R., Sikora P. (2021). Analysis of the impact of changes in thermomechanical properties of polymer materials on the machining process of gears. Polymers.

[6] Mehdipour-Ataei S., Tabatabaei-Yazdi Z. (2015). Heat Resistant Polymers. Encyclopedia of Polymer Science and Technology.

[7] Wilczyński K., Wilczyński K.J., Buziak K. (2022). Modeling and Experimental Studies on Polymer Melting and Flow in Injection Molding. Polymers.

[8] Tushar U., Jagtap H.A.M. (2015). Machining of Plastics: A Review. Int. J. Eng. Res. Gen. Sci..

[9] Karataş M.A., Gökkayab H. (2018). A review on machinability of carbon fiber reinforced polymer (CFRP) and glass fiber reinforced polymer (GFRP) composite materials. Def. Technol..

[10] Sheikh-Ahmad J.Y. (2009). Machining of Polymer Composites.

[11] Corrêa H.L., Rodrigues R.V., da Costa D.D. (2020). Machining process of glass-fiber-reinforced polyamide 6.6 Composite: Pathways to improve the drilling of recycled polymers. Eng. Res. Express.

[12] Balan A.S.S., Kannan C., Jain K., Chakraborty S., Joshi S., Rawat K., Alsanie W.F., Thakur V.K. (2021). Numerical modelling and analytical comparison of delamination during cryogenic drilling of cfrp. Polymers.

[13] Gaitonde V.N., Karnik S.R., Rubio J.C., Abrao A.M., Correia A.E., Davim J.P. (2011). Surface roughness analysis in high-speed drilling of unreinforced and reinforced polyamides. J. Compos. Mater..

[14] Soleymani Yazdi M.R., Razfar M.R., Asadnia M. (2011). Modelling of the thrust force of the drilling operation on PA6–nanoclay nanocomposites using particle swarm optimization. J. Eng. Manuf..

[15] Kuram E. (2016). Micro-machinability of injection molded polyamide 6 polymer and glass-fiber reinforced polyamide 6 composite. Compos. Part B.

[16] Yan Y., Mao Y., Li B., Zhou P. (2021). Machinability of the thermoplastic polymers: Peek, pi, and pmma. Polymers.

[17] Moghri M., Madic M., Omidi M., Farahnakian M. (2014). Surface Roughness Optimization of Polyamide-6/Nanoclay Nanocomposites Using Artificial Neural Network: Genetic Algorithm Approach. Sci. World J..

[18] Dhokia V.G., Kumar S., Vichare P., Newman S.T., Allen R.D. (2008). Surface roughness prediction model for CNC machining of polypropylene. J. Eng. Manuf..

[19] Raja Abdullah R.I., Yu Long A., Mohd Amran M.A., Kasim M.S., Mohd Hadzley A.B., Subramonian S. (2015). Optimisation of Machining Parameters for Milling Polyetheretherketones (PEEK) Biomaterial. Appl. Mech. Mater..

[20] Kumar J., Verma R.K., Mondal A.K., Singh V.K. (2021). A hybrid optimization technique to control the machining performance of graphene/carbon/polymer (epoxy) nanocomposites. Polym. Polym. Compos..

[21] Mata F., Petropoulos I.G., Ntziantzias J.P.D. (2010). A surface roughness analysis in turning of polyamide PA-6 using statistical techniques. Int. J. Mater. Prod. Technol..

[22] Madić M., Marinković V., Radovanović M. (2012). Mathematical modeling and optimization of surface roughness in turning of polyamide based on artificial neural network. Mechanika.

[23] Asghar A., Abdul Raman A.A., Daud W.M.A.W. (2014). A comparison of central composite design and Taguchi method for optimizing Fenton process. Sci. World J..

[24] Paulo Davim J., Reis P., Lapa V., Conceiçao António C. (2003). Machinability study on polyetheretherketone (PEEK) unreinforced and reinforced (GF30) for applications in structural components. Compos. Struct..

[25] Fountas N.A., Ntziantzias I., Kechagias J., Koutsomichalis A., Davim J.P., Vaxevanidis N.M. (2013). Prediction of Cutting Forces during Turning PA66 GF-30 Glass Fiber Reinforced Polyamide by Soft Computing Techniques. Mater. Sci. Forum.

[26] Aldwell B., Hanley R., O’Donnell G.E. (2014). Characterising the machining of biomedical grade polymers. J Eng. Manuf..

[27] Kaddeche M., Chaoui K., Yallese M.A. (2012). Cutting parameters effects on the machining of two high density polyethylene pipes resins: Cutting parameters effects on HDPE machining. Mech. Ind..

[28] Hamlaoui N., Azzouz S., Chaoui K., Azari Z., Yallese M.A. (2017). Machining of tough polyethylene pipe material: Surface roughness and cutting temperature optimization. Int. J. Adv. Manuf. Technol..

[29] Raj I.J.A., Vijayakumar P., Kannan T., Kumar P., Ragavan R.V. (2016). Design optimization of turning parameters of PTFE (Teflon) cylindrical rods using ANOVA Methodology. Int. J. Appl. Eng. Res..

[30] Chabbi A., Yallese M.A., Nouioua M., Meddour I., Mabrouki T., Girardin F. (2017). Modeling and optimization of turning process parameters during the cutting of polymer (POM C) based on RSM, ANN, and DF methods. Int. J. Adv. Manuf. Technol..

[31] Kilickap E., Huseyinoglu M., Yardimeden A. (2011). Optimization of drilling parameters on surface roughness in drilling of AISI 1045 using response surface methodology and genetic algorithm. Int. J. Adv. Manuf. Technol..

[32] Dadrasi A., Fooladpanjeh S., Gharahbagh A.A. (2019). Interactions between HA/GO/epoxy resin nanocomposites: Optimization, modeling and mechanical performance using central composite design and genetic algorithm. J. Braz. Soc. Mech. Sci. Eng..

[33] Davim J.P., Silva L.R., Festas A., Abrão A.M. (2009). Machinability study on precision turning of PA66 polyamide with and without glass fiber reinforcing. Mater. Des..

[34] Shahabaz S.M., Sharma S., Shetty N., Shetty S.D., Gowrishankar M.C. (2021). Influence of Temperature on Mechanical Properties and Machining of Fibre Reinforced Polymer Composites: A Review. Eng. Sci..

[35] Hazir E., Ozcan T. (2019). Response surface methodology integrated with desirability function and genetic algorithm approach for the optimization of CNC machining parameters. Arab. J. Sci. Eng..

[36] Antil P., Singh S., Kumar S., Manna A., Katal N. (2019). Taguchi and multi-objective genetic algorithm-based optimization during ecdm of sicp/glass fibers reinforced pmcs. Indian J. Eng. Mater. Sci..

[37] Janahiraman T.V., Ahmad N. (2018). Multi Objective Optimization for Turning Operation using Hybrid Extreme Learning Machine and Multi Objective Genetic Algorithm. Int. J. Eng. Technol..

